# Both 1-methylcyclopropene treatment and fresh-keeping mats mitigate peel browning and affect the aroma of Xinjiang “Yinggeer” flat peaches during cold storage

**DOI:** 10.1016/j.fochx.2025.103051

**Published:** 2025-09-20

**Authors:** Xiaobin Ji, Huijing Guo, Jiluan Chen, Fangyuan Song

**Affiliations:** a Institute of Agricultural Products Processing, Xinjiang Academy of Agricultural Reclamation Sciences, Shihezi, Xinjiang 832000, China; b Food College, Shihezi University, Xinjiang 832003, China

**Keywords:** Fruit, Peel browning, Path analysis, Physiological quality, Aroma volatiles

## Abstract

The current study explored the effects of 1-methylcyclopropene (1-MCP) and fresh-keeping mats (FKP) on aroma volatile levels and peel browning (PB) in flat peaches during.

cold storage. Both 1-MCP and FKP treatments prevented PB by inhibiting the activity of polyphenol oxidase, lipoxygenase, and peroxidase; reducing relative electrolytic leakage; and down-regulating malondialdehyde levels, thus increasing the content of unsaturated fatty acids. Although both the 1-MCP and FKP treatments prevented PB, FKP-treated fruit showed a higher content of aromatic compounds, greater fruit firmness, delayed reduction in the soluble solids content, and lower titratable acidity. Correlation and pathway analysis demonstrated that PB was positively correlated with polyphenol oxidase activity, peroxidase activity, relative electrolytic leakage, and malondialdehyde levels. Notably, the reduced peroxidase and polyphenol oxidase activities, relative electrolytic leakage, and malondialdehyde content inhibited PB in flat peaches. These findings show that FKP treatment is a simple and effective strategy for improving the storage quality of flat peaches.

## Highlights

• Treatments alleviated PB by inhibiting related enzymes activities.
• PPO activity was the most significant factor affecting PB.
• JZ and 1-MCP treatments inhibited PB increase.
• JZ-treated had more aromatic compounds.
• JZ-treated maintained good refrigeration quality of flat peaches.

## 1 Introduction

Shihezi in Xinjiang, China, is recognized as the hometown of the flat peach. Among flat peaches, the “Yinggeer” variety is highly favored by consumers owing to its rich nutritional components and unique flavor. Flat peaches not only have a thin pericarp but also show climacteric features and high chilling sensitivity (Song et al., 2024). As a result, they tend to perish during storage and transportation, which limits their availability in certain regions. Post-harvest peach fruit becomes soft within 2–3 d at ambient temperature (Xi et al., 2012) and exhibits pericarp browning. Its soluble solids content (SSC) and titratable acidity (TA), essential for the sweet-sour taste balance, also undergo alterations. These alterations, along with changes in aroma—a key factor influencing consumer purchasing decisions—affect the overall quality of the fruit. To extend the postharvest life of fruit, cold storage is extensively utilized. However, cold storage can easily cause chilling injury (CI), which manifests as peel browning (PB), diminished aroma, as well as woolliness and leatheriness (Franzoni et al., 2023). In particular, PB is a key indicator of CI in flat peaches (Liu, Jiang, et al., 2019) and significantly affects their marketability and shelf life. Various physical post-harvest treatments, such as controlled atmosphere (Zhou et al., 2022), and chemical treatments, such as salicylic acid (Zhao et al., 2021) and 1-methylcyclopropene (Zhang, Shao, et al., 2020), are used to prevent CI in fruit.

Previous studies have indicated that the balance between unsaturated and saturated fatty acids, along with membrane lipid peroxidation and subsequent degradation, contributes to fruit browning (Zhang & Tian, 2010). In particular, cell membrane degradation is correlated with the activity of lipoxygenase (LOX) (Singh et al., 2012), which destabilizes cell membranes (Gao et al., 2017). Cell membrane damage is the primary event in PB (Kou et al., 2015) and is mainly caused by the reaction of phenols with oxygen to produce ortho-quinones, which is mediated by polyphenol oxidase (PPO) (Lin et al., 2014). Another contributor to PB is the lipid peroxidation product malondialdehyde (MDA), whose generation can be inhibited by peroxidase (POD) and catalase (CAT), which are important free radical scavenging antioxidant enzymes in plant tissues(Xie et al., 2022).

In addition to PB, the content of aromatic substances is also an important indicator of fruit quality (Liu, Liu, et al., 2019). Interestingly, the deterioration of fruit aroma, particularly the reduction of C6 aldehydes/alcohols and lactones, is associated with flesh browning (Li et al., 2023). So far, more than 100 aroma volatiles have been detected in peach fruit. However, only a few components and representative characteristic compounds determine the aroma of this fruit (Maoz et al., 2023). As mentioned previously, 1-MCP is widely used to preserve fruit aroma (Sun et al., 2019) and prevent CI in different types of fruit, such as nectarines (Zhang et al., 2020a, Zhang et al., 2020b). However, the mechanisms underlying its effects on CI, PB, and aroma compounds in flat peach remain unexplored, especially in comparison to the effects of physical treatments like fresh-keeping mats. Fresh-keeping mats containing 25 % sodium metabisulfite and 0.33 % 1-MCP. Notably, sodium metabisulfite can achieve preservation, anti-oxidation, and anti-browning effects by slowly releasing sulfur dioxide. Meanwhile, 1-MCP is an ethylene absorber. By combining these two agents, the post-harvest storage quality of horticultural crops can be maintained, and their storage period can be extended (Sun et al., 2019).

Therefore, the current study explored how 1-MCP and fresh-keeping mats affect PB, aroma volatiles, membrane fatty acid levels, relative electrolyte leakage, MDA levels, and LOX, POD, PPO, and CAT activities in flat peaches during cold storage. Moreover, the correlations among MDA levels (X1), relative electrical leakage (X2), CAT activity (X3), POD activity (X4), PPO activity (X5), LOX activity (X6), palmitic acid levels (X7), stearic acid levels (X8), oleic acid levels (X9), linoleic acid levels (X10), linolenic acid levels (X11), and elaidic acid levels (X12) were analyzed in flat peaches subjected to 1-MCP and fresh-keeping mat treatments. Finally, path analysis was used to explore the effects of these 12 factors on PB in flat peaches. This study aims to provide a theoretical and practical basis for the further exploration of CI mechanisms in fruit, enabling the development of new methods for extending fruit shelf life and preventing fruit browning during refrigeration.

## 2 Materials and methods

### 2.1 Sample handling

At eight-degree maturity (SSC > 12 %, firmness >5), flat peaches were harvested from the orchard of Shihezi city, Xinjiang Province, China. Fruit of uniform size and color and free from pests and diseases were harvested on July 29, 2024. The use of 0.5 g 1-MCP was based on the optimal dosage determined in our previous experiments, which evaluated the effects of different dosages on the storage quality of flat peaches. The fruit were placed in plastic frames lined with a blowing net and immediately transported to a phase-temperature reservoir. Subsequently, the fruit were subjected to fresh-keeping mat treatment (FKP treatment group), subjected to 1-MCP (0.5 g) treatment (1-MCP treatment group), or left untreated(CK group) during cold storage (0 ± 0.5 °C, relative humidity: 85 ± 5 %). Notably, there were 400 fruit in each group. Sampling was performed at 5, 10, 15, 20, 25, and 30 d, with three biological replicates consisting of 20 fruit each being used for analysis per time point. The fruit samples were cut into small pieces, frozen in liquid nitrogen, and stored at −80 °C for further analysis.

### 2.2 Physiological parameters

Flat peach firmness was determined using a tester (GY-4, Zhejiang Handpi Instrument Co. Ltd., Le Qing, China)fitted with a flat probe (2 mm in diameter), and three replicates of 10 fruits each were tested per group. The unit of fruit firmness was N. Subsequently, a handheld saccharometer (WZ 108, TOP Instrument Co. Ltd., Zhejiang, China) was used to measure the SSC of flat peaches, with three replicates of 10 fruits each per group. TA was measured according to the method described by Tian et al. (2020). To this end, 20 g tissues from 30 fruits were pooled and used for each evaluation. Both the SSC and TA were expressed as% values.

### 2.3 Analysis of relative electrolyte leakage, MDA levels, and PB

The value of relative conductivity was measured according to the method described by Song et al. (2024), with three replicates of 10 fruits per group (15 g each). Fresh fruit samples were cut into 1-mm pieces and immersed in 40 mL of deionized water before measuring relative electrolyte leakage using a conductivity meter (DDS-307, Yidian Thunder Magnetic Co., LTD., Shanghai, China). The initial electrolyte leakage was recorded as P_0_, and the electrolyte leakage after 10 min was expressed as P_1_. Subsequently, P_2_ was measured after boiling the samples for 10 min and cooling to 25 °C. The calculation formula is as follows:

Relativeelectrolyteleakage%=P1−P0P2−P0×100%

Subsequently, the thiobarbituric acid (TBA) method was used to measure the content of MDA, with three replicates of 10 fruits per group (0.5 g each) as described by Zhang, Shao, et al. (2020), and the results were expressed as μ mol·kg^−1^. Meanwhile, PB was evaluated using 20 fruits per group following the method reported by Liu, Jiang, et al. (2019) based on the area of browning and expressed as a percentage (%) value.

### 2.4 Analysis of enzyme activity

LOX activity was determined according to a previously reported method (Hu et al., 2022). In total, 20 fruits were analyzed per group. First, 1 g of flat peach tissue frozen using liquid nitrogen was ground into a fine powder and blended with 5 mL of 50 mmol·L^−1^ phosphate extraction solution (pH 7.0). Then, 10 mmol·L^−1^ sodium soyate was used as the substrate, and 0.2 mL of crude enzyme was added to a 2.7 mL phosphate buffer system.

PPO and POD activity were measured by referring to the method described by Buitimea-Cantúa et al. (2023) and 20 fruits were examined per group. CAT activity was determined based on the protocol reported by Hu et al. (2014) using 20 fruits per group. First, 5 g of each sample was homogenized on ice with 5 mL of 50 mmol·L^−1^ sodium acetate buffer (pH 5.6). The mixture was then centrifuged at 12,000 ×*g* and 4 °C for 30 min. The reaction mixture consisted of 2.9 mL 20 mmol·L^−1^ H_2_O_2_ and 0.1 mL enzyme solution. The activity of CAT was calculated as 0.01 ΔOD_240 nm_ per minute per gram of enzyme extract under standard reaction conditions. The unit for LOX, PPO, POD, and CAT activity based on the fresh weight of flat peaches was U·kg^−1^.

### 2.5 Fatty acid analysis

To initiate the extraction, 1 g of liquid nitrogen-frozen flat peach tissue was homogenized and then combined with 10 mL of a petroleum ether/diethyl ether mixture (4:3, *v*/v). The mixture was incubated at 4 °C for 24 h. Following this, 10 mL of a 0.4 mol·L^−1^ potassium hydroxide-methanol solution was introduced to the mixture for a 2-h methyl esterification at 25 °C. The reaction solution was subsequently centrifuged at 66.67 r·s^−1^ for 10 min at 25 °C. Finally, a 5 mL aliquot of the upper organic layer, containing the lipids, was transferred to a 10 mL nitrogen blowpipe and evaporated to dryness under a stream of nitrogen gas. The residue obtained was mixed with 1 mL hexane and stored at −20 °C for subsequent gas phase analysis. Qualitative analysis was performed using 37 mixed standards of fatty acid methyl esters. The unit of fatty acid content was g·kg^−1^.

### 2.6 Measurement of aroma compounds using HS-SPME-GC–MS

The analysis of volatile compounds in flat peach was conducted based on the method of Tian et al. (2017) employing headspace solid-phase microextraction coupled with gas chromatography–mass spectrometry (HS-SPME–GC–MS). Each experimental group consisted of 20 fruits. Prior to analysis, the SPME fiber was conditioned at 250 °C for 30 min. Specifically, 7 g of powdered sample was weighed into a 20 mL headspace vial, mixed with 3 mL of saturated sodium chloride solution and 10 μL of 2-octanol (internal standard, 0.00544 g·L^−1^), and then incubated at 50 °C for 5 min. A 65-μm polydimethylsiloxane/divinylbenzene (PDMS/DVB) extraction head was inserted into the sample vial, and solid phase micro-extraction (SPME) sampling was performed for 46 min at 50 °C. After the SPME sampling process, the fiber was immediately inserted into the gas chromatography–mass spectrometry (GC–MS) inlet. A 6890-gas chromatograph with SPME coupled with a 5973-mass selective detector was used for analyzing volatile compounds. A DB-WAX capillary column (30 m × 0.25 mm × 0.25 um, Agilent 122–7032) was employed for separating the compounds. Helium (99.999 %) was used as the carrier gas and was continuously injected at a flow rate of 0.017 mL·s^−1^. The initial temperature was 40 °C for 3 min; it was increased to 90 °C at a rate of 0.1 °C·s^−1^ and then to 230 °C at a rate of 0.167 °C·s^−1^. Finally, the temperature was maintained at 230 °C for 7 min. The MS settings used by Song et al. (2024) were employed. Aroma compounds were identified using the NIST17 library (≥ 80 %), and the content of aroma compounds was calculated using internal standards. The contents of aroma compounds were expressed as mg·kg^−1^.

### 2.7 Statistical analysis

All values are expressed as the mean ± standard deviation (SD) of three replicates. Duncan's multiple range test was used to determine inter-group differences. Statistical analyses and pathway analyses were performed using SPSS software version 22 for Windows (SPSS Inc., Chicago, IL). X1, X2, X3, X4 X5 X6, X7, X8, X9, X10, X11, and X12 were all independent variables, and the degree of PB served as the dependent variable. The determination coefficients for both two-factor and single-factor analyses, along with indirect effects, were calculated using correlation coefficients and direct path coefficients (Zhao et al., 2018). Heatmaps were generated using Omic Studio tools to visualize differences in volatile compounds. Figures were generated using Origin Pro 2022 software (OriginLab Corporation, USA).

## 3 Results

### 3.1 FKP and 1-MCP treatment affect quality and PB in flat peach

**Fig. 1:**
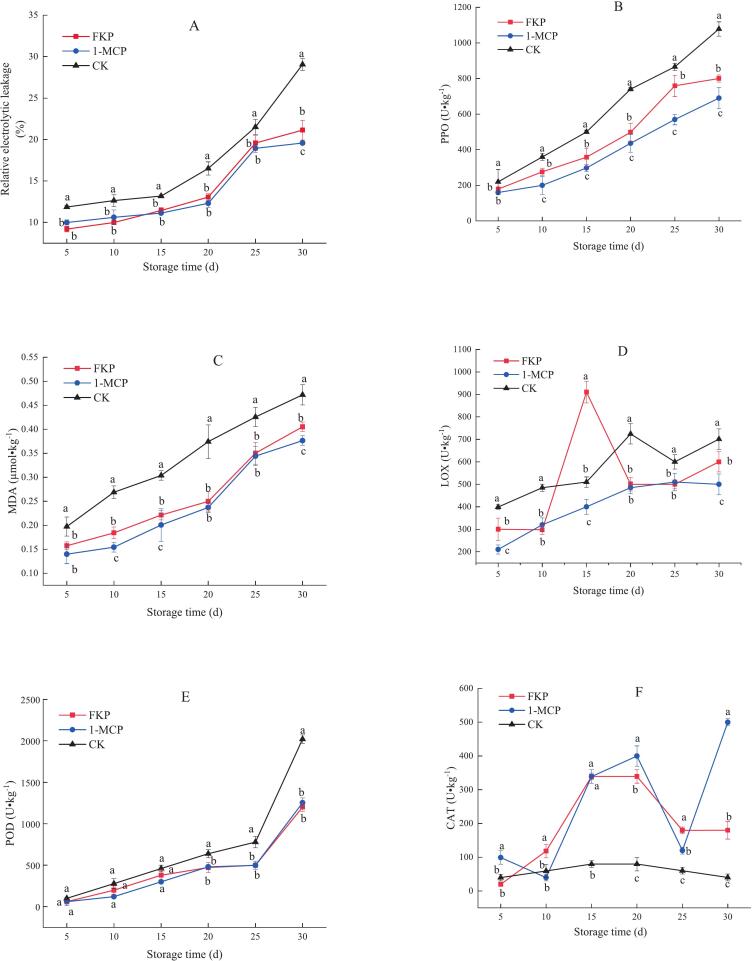
Changes in fruit firmness (A), PB index (B), SSC (C), and TA (D) in the FKP and 1-MCP treatment groups of flat peaches stored at 0 ± 0.5 °C. The values are presented as the mean ± SD (*n* = 3). Statistical significance (*P* < 0.05) between treatments at a given time point is indicated by different letters.

1-MCP treatment was marginally better than FKP treatment at preventing fruit softening in flat peaches (Fig. 1A). The loss of fruit firmness in the CK group was mainly observed during the first 20 d of storage. PB is an indicator of CI severity. In the current study, PB was evaluated based on the area of peel browning. Notably, no PB was observed in any group during the first 10 d of storage(Fig. 1B). However, PB was detected in “Yinggeer” flat peaches after day 10, showing a significant increase. However, no differences in PB indices were observed among the different treatment groups beyond this time point. The PB index was similar between the FKP and 1-MCP treatment groups between days 10 and 30. However, both the FKP and 1-MCP treatment groups exhibited significantly lower PB indices than the CK group.

Interestingly, the SSC values of flat peaches subjected to FKP and 1-MCP treatments increased gradually during the storage period (Fig. 1C). Overall, the SSC in the CK group showed an increasing trend between days 5 and 30, although a slight decrease was noted on day 15. The content of SSC in the CK group was higher than that in the FKP and 1-MCP treatment groups. Meanwhile, on day 30, the highest TA was observed in the 1-MCP treatment group, followed by the FKP and CK groups (Fig. 1D).

### 3.2 Influence of different treatments on enzyme activity, MDA levels, and relative electrolytic leakage in flat peach

**Fig. 2:**
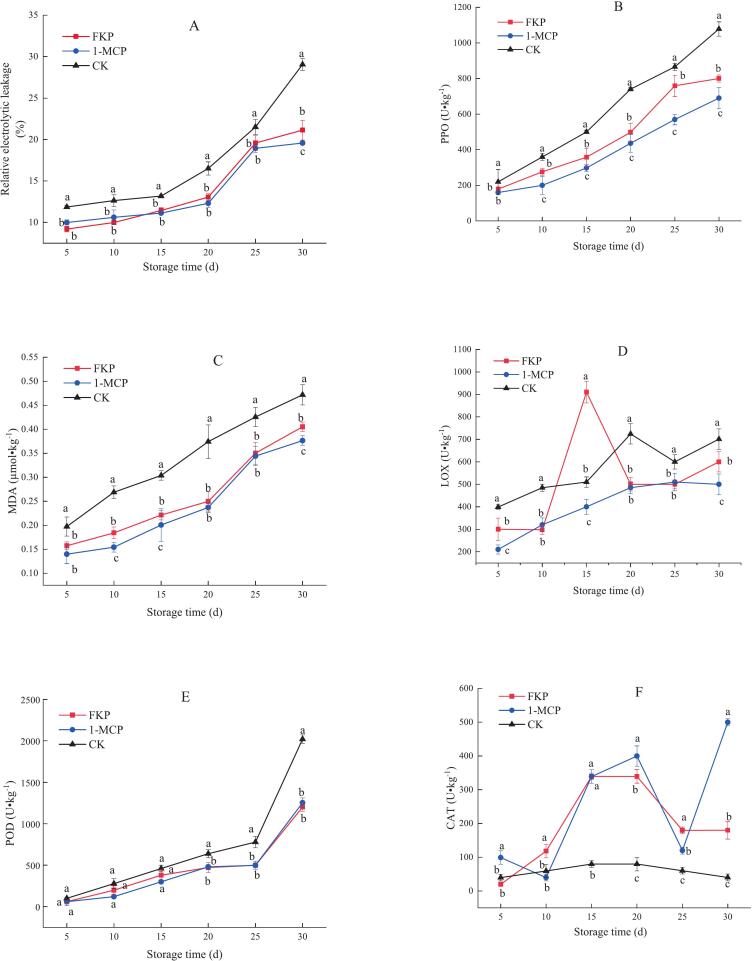
FKP and 1-MCP treatment affects relative electrolytic leakage (A), PPO activity (B), MDA content (C), LOX activity (D), POD activity (E), and CAT activity (F) in flat peaches stored at 0 ± 0.5 °C. The values are presented as the mean ± SD (*n* = 3).

As shown in Fig. 2A, the relative electrolytic leakage in the fruit of all treatment groups increased throughout storage. The CK group showed higher relative electrical conductivity than the 1-MCP and FKP groups throughout the storage period. The relative electrical conductivity in the FKP group was slightly higher than that in the 1-MCP group during the later stage of storage. On day 30, the relative electrolytic leakage in the CK group was 7.9 % and 9.5 % higher than that in the FKP and 1-MCP treatment groups, respectively.

Throughout the storage period, the activity of PPO in the CK group remained higher than that in the 1-MCP and FKP treatment groups. Furthermore, plat peaches treated with 1-MCP showed lower PPO activity than those receiving FKP treatment (Fig. 2B). Meanwhile, the variations in MDA content were consistent with the trends of PPO activity across the three groups. The content of MDA increased gradually with storage time in all treatment groups(Fig. 2C). Notably, the MDA content in the 1-MCP and FKP treatment groups was lower than that in the CK group during cold storage.

No difference in LOX activity was observed between the FKP and 1-MCP treatment groups throughout the storage period, except on day 15. The LOX activity in all treatment groups increased gradually with prolonged storage. The LOX activity in the FKP treatment group increased during the first 15 d of storage, decreased slightly on day 20, and then increased toward the end of the storage period (Fig. 2D). Meanwhile, fruit treated with FKP and 1-MCP showed no differences in POD activity. Notably, the POD activity of fruit treated with FKP and 1-MCP was lower than the POD activity in the CK group on day 30 (Fig. 2E). Although CAT activity was comparable between the FKP and 1-MCP treatment groups on days5–20, a difference was observed between days 25 and 30. The CAT activity initially increased in the two treatment groups before decreasing thereafter. In the CK group, the CAT activity showed a slight increase at first and then tended to decrease as storage was extended. Notably, the 1-MCP treatment group showed the highest CAT activity, followed by the FKP treatment group. In contrast, the CK group showed the lowest CAT activity (Fig. 2F).

### 3.3 Influence of different treatments on the content of fatty acids in flat peach

**Fig. 3:**
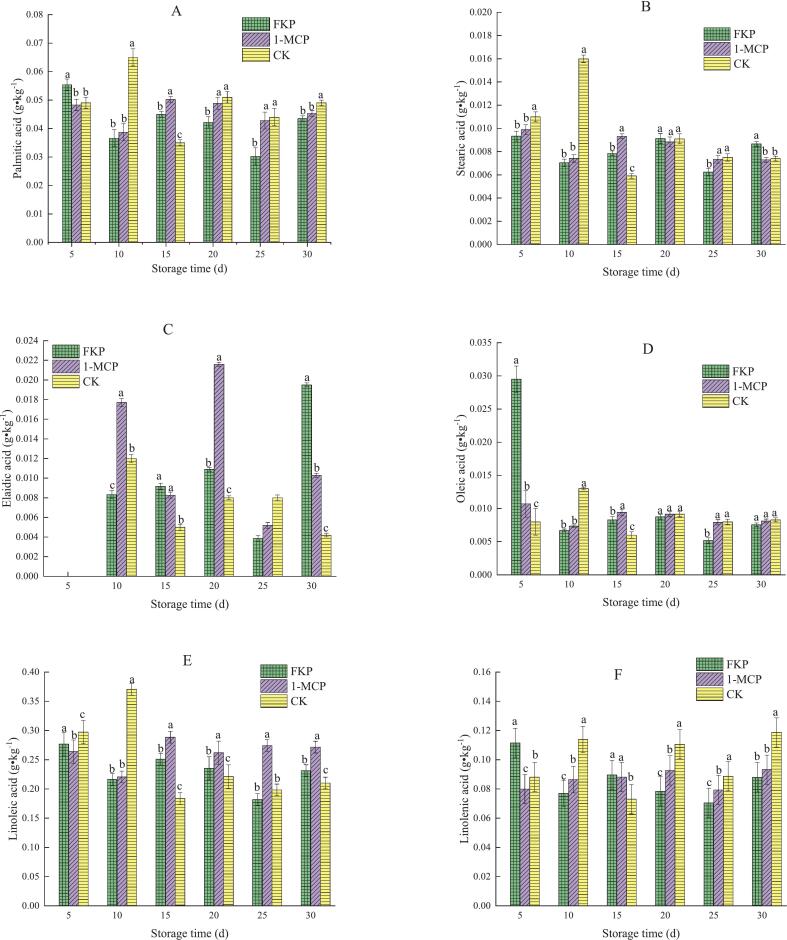
Effects of FKP and 1-MCP treatment on the contents of palmitic acid (A), stearic acid (B), elaidic acid (C), oleic acid (D), linoleic acid (E), and linolenic acid (F) of flat peaches stored at 0 ± 0.5 °C. The values are presented as the mean ± SD (*n* = 3).

Six major membrane fatty acids were detected in “Yinggeer” flat peaches, including two saturated fatty acids and four unsaturated fatty acids. Specifically, the palmitic acid content showed a similar trend in the three treatment groups between day 20 and day 30, with an initial decrease followed by an increase (Fig. 3A). The content of stearic acid in FKP- and 1-MCP-treated fruit showed a similar trend between day 5 and day 25. No difference in stearic acid levels was observed between the 1-MCP and CK group on day 30(Fig. 3B). Elaidic acid was not detected in flat peaches during the first five days of storage (Fig. 3C). The levels of stearic and elaidic acid in FKP-treated fruit were higher than those in the 1-MCP-treated and untreated fruit. Oleic acid showed 34 % higher levels in FKP-treated fruit on day 5 versus day 10 (Fig. 3D).

The content of linoleic acid was higher in the FKP and 1-MCP treatment groups than in the CK group between day 15 and day 30 (Fig. 3E). The linoleic acid content in 1-MCP-treated fruit was slightly higher than that in FKP-treated fruit. Between day 5 and day 20, the changes in linoleic acid content were similar in the 1-MCP and FKP-treated fruit, but the opposite trend was observed between day 20 and day 25. The linolenic acid content in 1-MCP-treated fruit increased between day 5 and day 20. However, in fruit treated with FKP, the linolenic acid content first declined, then increased, and finally decreased again during this period (Fig. 3F).

### 3.4 Effect of FKP and 1-MCP treatment on aroma compounds in flat peach

**Fig. 4:**
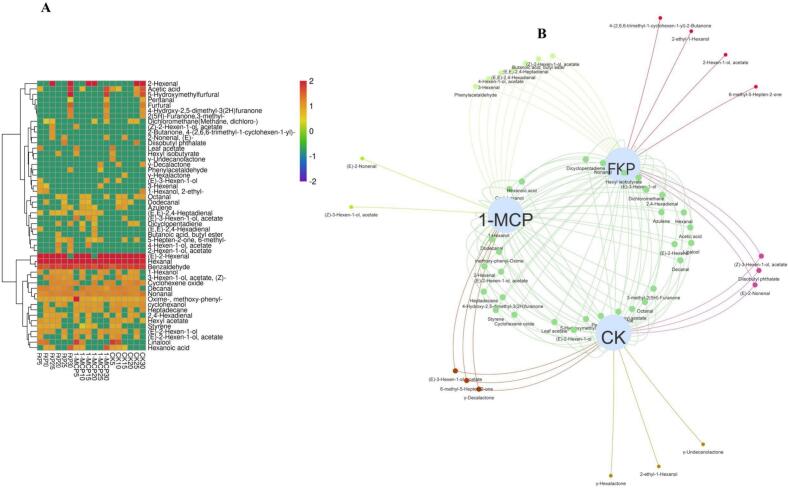
Effect of FKP and 1-MCP treatment on the levels of 50 aroma compounds in flat peaches stored at 0 ± 0.5 °C. (A) Heatmap of aroma compounds. (B) Network diagram of aroma compounds.

Fifty volatile compounds were identified in “Yinggeer” flat peach and quantified, including 17 aldehydes, 11 esters, six alcohols, four ketones, three lactones, 3 alkenes, two acids, one alkane, and three other compounds. The variations in the volatile compounds during storage are shown in Fig. 4A. Cluster analysis using the ward. D2 method resulted in a three-cluster solution for the 50 volatile compounds. (E)-2-hexenal, hexanal, and benzaldehyde were grouped into the first cluster, and 1-hexanol (Z)-3-hexen-1-ol, acetate, cyclohexene oxide, decanal, nonanal, oxime-, methoxy-phenyl-, cyclohexanol, heptadecane, 2,4-hexadienal, hexyl acetate, styrene, (E)-2-hexen-1-ol, (E)-2-hexen-1-ol, acetate, linalool, and hexanoic acid were assigned to the second; and cluster 3 contained all other identified aroma compounds. Of these, (E)-2-hexenal, hexanal, benzaldehyde, and 2-hexenal exhibited the highest total concentrations. Hence, cluster 1 compounds showed the highest concentration, followed by cluster 2 compounds, while cluster 3 compounds had the lowest concentrations.

### 3.5 Correlation analysis of indices associated with PB in the FKP and 1-MCP groups

**Fig. 5:**
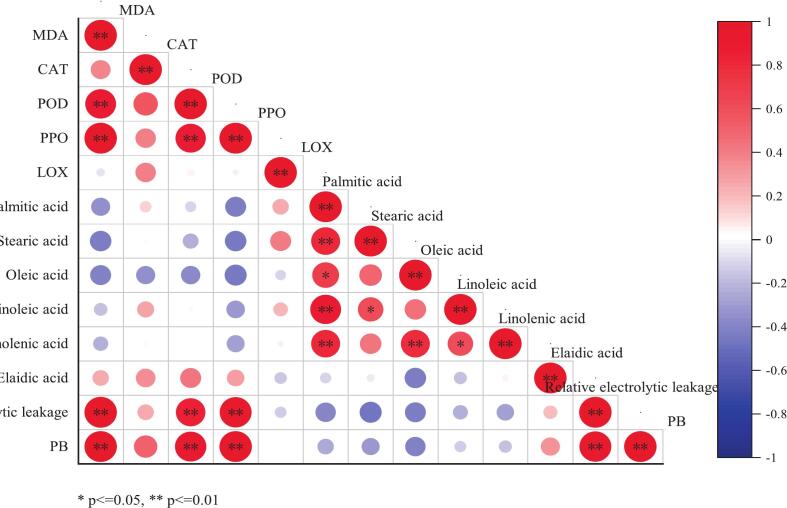
Pearson's correlation coefficients of factors related to PB in flat peaches stored at 0 ± 0.5 °C after FKP treatment (A) and 1-MCP treatment (B). * indicates difference between the two groups at *P* < 0.05; ** indicates significant difference between two groups at *P* < 0.01.

Pearson correlation analysis was performed to examine the relationships among membrane permeability, MDA levels, antioxidant enzyme (POD and CAT) activity, PPO activity, LOX activity, fatty acid levels, and PB in FKP- and 1-MCP-treated flat peaches across different storage time points. Pearson correlation coefficients with an absolute value closer to 1 indicate a stronger correlation (Wei et al., 2018). In this study, PB in FKP-treated fruit was found to be positively correlated with POD activity, PPO activity, relative electrolytic leakage, and MDA levels (Fig. 5A). The strength of correlations was as follows: PPO (*r* = 0.98) = relative electrolytic leakage (*r* = 0.98) > MDA (*r* = 0.93) > POD (*r* = 0.91). Linolenic acid levels showed a strong positive correlation with oleic acid, linoleic acid levels and palmitic. Further, MDA levels, relative electrolytic leakage, elaidic acid levels, PPO activity, and POD activity were all positively correlated with each other. These findings indicated that low POD and PPO activities, relative electrolytic leakage, and MDA content inhibited PB in flat peaches during cold storage.

### 3.6 Path analysis of indices associated with PB in the FKP and 1-MCP groups

Path analysis was performed to further explore the major determinants of PB during cold storage in FKP- and 1-MCP-treated “Yinggeer” flat peaches. Subsequently, the absolute value of path coefficients (i.e., the sum of direct and indirect effects) was used to determine the importance of each factor in the context of PB. As shown in Table 1, X5 was the independent variable that showed the strongest correlation with PB in FKP-treated fruit, followed by X10 and X6. Of these, X5 exerted the greatest direct effects on PB, followed by X10 and X6. However, X10 exerted the greatest indirect effects on PB, followed by X6 and X5. By comparing the absolute values of the determination coefficients for PB, we found that PPO activity was the most important factor affecting PB in FKP-treated fruit, followed by the interaction between linoleic acid level and PPO activity, linoleic acid levels, the interaction between LOX and PPO activity, the interaction between LOX activity and linoleic acid levels, and LOX activity. The impact of other factors (not shown in Table 1) was minimal.

**Table. 1:** Path analysis of physiological factors affecting PB after FKP treatment.

Factor (X)	Path coefficients with PB	Direct Effects	Indirect Effects			
			X5	X6	X10	Total Indirect Effects
X5	0.981	1.124	–	0.010	−0.153	−0.143
X6	0.359	0.039	0.298	–	0.022	0.320
X10	−0.492	0.236	−0.732	0.004	–	−0.728

As shown in Table 2, X5 showed the highest path coefficients in 1-MCP-treated fruit, followed by X1, X3, X8, and X9. Of these, X5 exerted the greatest direct effects on PB, followed by X3, X9, X1, and X8, whereas X1 exerted the greatest indirect effects on PB, followed by X3, X8, X9, and X5. Based on the determination coefficients for PB, PPO activity emerged as the most important factor influencing PB in 1-MCP-treated fruit, followed by MDA levels, CAT activity, stearic acid levels, the interaction between CAT and PPO activity, and oleic acid levels. The interaction effect of CAT activity and stearic acid levels had the least influence on PB. The remaining factors with little influence on PB are not shown in Table 2.

**Table. 2:** Path analysis of physiological factors affecting PB after 1-MCP treatment.

Factor (X)	Path coefficients with PB	Direct Effects	Indirect Effects					
			X1	X3	X5	X9	X8	Total Indirect Effects
X1	0.956	−0.025	–	0.091	0.912	−0.023	0.001	0.981
X3	0.719	0.132	−0.016	–	0.600	0.003	0	0.587
X5	0.993	0.952	−0.023	0.083	–	−0.020	0.001	0.041
X9	−0.351	0.047	0.012	0.008	−0.416	–	−0.002	−0.398
X8	−0.530	−0.002	0.016	−0.001	−0.587	0.044	–	−0.528

## 4 Discussion

Firmness, SSC, and TA are important indices for evaluating fruit quality. The fruit in both the FKP and 1-MCP treatment groups maintained their firmness during the storage period. Previous studies have shown that 1-MCP can delay softening in various types of fruit by inhibiting ethylene release (Ozkaya et al., 2016; Liu et al., 2018). In this study, fruit firmness in the 1-MCP group was approximately 37 % higher than that in the CK group on day 30. Moreover, our analysis showed that a higher PB index was correlated with reduced fruit firmness in flat peaches. The relatively high loss of SSC in the CK group could be attributed to the deterioration of fruit quality due to CI and high respiration rates. These findings are consistent with previous reports showing that 1-MCP treatment can delay SSC reductions (Chen et al., 2018). Similarly, previous studies have reported that 1-MCP can prevent CI in peaches and nectarines (Liu et al., 2018; Zhang, Shao, et al., 2020). Moreover, we found that the TA values of flat peach fluctuated significantly between day 5 and day 30. However, the TA values decreased with fruit ripening (Sun et al., 2019), likely due to the consumption of organic acids during respiration (Pushpendra & Shruti, 2017). Moreover, in this study, the PB index in the CK group on day 30 was 9.4 %, implying that the incidence of CI was relatively low in flat peaches stored at 0 °C, consistent with studies on other fruit (Song et al., 2022).

LOX is involved in membrane lipid metabolism, where it catalyzes the conversion of UFA to SFA and induces membrane lipid peroxidation (Zhang et al., 2018).The LOX activity in the FKP and 1-MCP treatment groups was inhibited when compared to that in the CK group throughout the storage period in the present study. Low LOX activity can delay membrane lipid peroxidation, protecting the structural integrity of cell membranes and prolonging the shelf life of fruit. These alterations manifest as reductions in MDA levels and relative electrolytic leakage, which are important for determining the degree of membrane lipid peroxidation and cell membrane permeability, respectively, and are used to indirectly evaluate membrane integrity (Aghdam & Bodbodak, 2013). Our findings are in line with previous results showing that treatment with 1-MCP and fresh-keeping mats decreases the MDA content and relative electrolytic leakage (Lin et al., 2018; Liu, Jiang, et al., 2019; Phetsirikoon et al., 2012; Ran et al., 2012) in fruit.

Studies report that an elevated PB index, which signifies an increase in fruit discoloration, is mainly caused by the oxidation of phenolic compounds under the catalytic action of the enzyme PPO (Navarro et al., 2024). Notably, pulp browning due to PPO-mediated oxidation is a major problem in refrigerated peach fruit (Zhang et al., 2022). A high PB index is an external manifestation of pulp browning during fruit ripening under storage. In this study, the trends of PPO and POD activity were found to be similar, suggesting that FKP and 1-MCP treatment inhibited both PPO and POD activity during storage. The PB index in FKP- and 1-MCP-treated fruit was lower than that in the CK group, consistent with findings from previous studies (Ozkaya et al., 2016; Gao et al., 2017). Moreover, 1-MCP treatment was more effective at attenuating increased PB and maintaining fruit quality than FKP treatment. Similarly, a previous study showed that 1-MCP treatment increases CAT activity in kiwi fruit (Wang et al., 2014). These findings collectively indicate that FKP and 1-MCP treatment reduce MDA accumulation in flat peaches and prevent PB, likely by inhibiting PPO, POD, and LOX activities and increasing CAT activity, consistent with previous studies (Gao et al., 2017; Wang et al., 2019).

In total, 10 fatty acids were identified in all stored fruit samples, including six saturated fatty acids (lauric acid, palmitic acid, perlitic acid, stearic acid, arachidic acid, and lignoceric acid) and four unsaturated fatty acids (elaidic acid, oleic acid, linoleic acid, and linoleic acid). Linoleic acid was the primary membrane lipid UFA, followed by linolenic acid. In contrast, previous studies have identified linolenic acid as the main UFA in fruit membranes (Wang et al., 2019; Zhang & Tian, 2009). These differences are likely the result of factors such as variations in fruit type and growth conditions. The fatty acid profile of flat peach identified in the current study was similar to that of “pomegranate skin,” as reported previously (Gp et al., 2020). Notably, linoleic acid and palmitic acid showed the highest levels among the different types of UFA and SFA, respectively. Peng et al. (2014) reported that a higher UFA/SFA ratio is correlated with a lower degree of browning. In the current study, the PB index of flat peaches in the 1-MCP and FKP groups was lower than that in the CK group. This demonstrated that the two treatments could delay the occurrence of PB in flat peaches by regulating the fatty acid content.

Interestingly, the levels of aromatic compounds detected in the present study were lower than previously reported levels (Zhou et al., 2018). A network diagram was constructed for further intuitive analysis to compare the types and quantities of aromatic compounds in the CK, FKP, and 1-MCP treatment groups (Fig. 4B).The concentration of volatile compounds in FKP-treated fruit was higher compared to that in the 1-MCP-treated and untreated groups. The FKP group showed the highest total content of volatile substances between day 5 and day 30, followed by the CK group. In contrast, the 1-MCP treatment group exhibited the lowest content of volatile compounds. This implies that 1-MCP treatment inhibited the production of aromatic compounds in fruit, as evidenced in previous studies (Zhang et al., 2014). The content of volatile compounds in flat peaches detected in the present study was lower than previously reported levels in fresh flat peach juice (Wang et al., 2020), likely because the juice was more concentrated, while the content of volatile compounds was higher.

Aldehydes and esters are responsible for grassy and fruity aromas in fruit, respectively, and give fruit a pleasant taste. Thus, their content is an important indicator of flavor quality. In this study, FKP-treated fruit showed higher aldehyde and ester levels and lower alcohol levels than 1-MCP-treated fruit. Seven compounds were common to the FKP and 1-MCP groups, namely, phenylacetaldehyde, (E, E)-2, 4-hexadienal, (E, E)-2, 4-heptadienal, 3-hexenal, butanoic acid, butyl ester, 4-hexen-1-ol, acetate, and (Z) -2-hexen-1-ol, acetate. These findings indicate that FKP treatment led to slightly better flavor quality than 1-MCP treatment.

The PB index in 1-MCP-treated fruit showed strong positive correlations with POD, PPO, and LOX activity; MDA content; and relative electrolytic leakage (*r* = 0.91) (Fig. 5B). This was in line with previous findings (Cao et al., 2010) showing CI prevention following 1-MCP treatment, accompanied by reduced LOX activity. Moreover, our study found positive correlations among MDA levels, relative electrolytic leakage, LOX activity, POD activity, and PPO activity. Furthermore, a positive correlation was detected between CAT activity and linolenic acid levels, while LOX activity showed a weak positive correlation with POD activity and relative electrolytic leakage. Previously, Gao et al. (2017) demonstrated that low PPO and POD activities were responsible for reduced flesh browning in peach fruit. Similar results were observed in the present study, which found that low LOX, POD, and PPO activities; high CAT activity; low relative electrolytic leakage; low MDA levels; and high linolenic acid levels prevent PB. Through further analyses, the strongest correlation was observed between POD activity and MDA levels in the two treatment groups. Specifically, PB demonstrated a distinct positive correlation with MDA levels and relative electrolytic leakage, which is consistent with a previous report (Babalar et al., 2018). Collectively, these findings show that different treatments influenced the degree of PB in flat peaches during storage. PPO activity, POD activity, MDA content, and relative electrolytic leakage were all positively correlated with PB in the FKP and 1-MCP treatment groups.

Fruit discoloration is primarily caused by the oxidation of phenolic substrates via PPO. Thus, higher PPO activity contributes to internal browning in peaches, apples, and pears fruit stored at 0 °C (Peng et al., 2009; Prange & Wright, 2023). In this study, path analysis in the two treatment groups showed that PPO activity was the most critical factor affecting PB, in line with previous reports. Although this study demonstrates that 1-MCP and fresh-keeping mats are effective at inhibiting the post-harvest browning of flat peaches to a certain degree, it has certain limitations with regard to the comprehensive evaluation of flat peach shelf life and flavor metabolism. Future research should focus on the influence of fresh-keeping technology on the storage quality of flat peaches and their shelf life, so as to provide scientific guidance for the development of improved post-harvest fresh-keeping technologies.

## 5 Conclusion

In summary, treatment with FKP and 1-MCP can effectively alleviate PB in flat peaches by reducing PPO, POD, and LOX activity; MDA levels; and relative electrolytic leakage and increasing CAT activity. Notably, PPO activity emerged as the primary determinant of PB in flat peaches. Path analysis after FKP treatment revealed that the interaction between linoleic acid levels and PPO activity and linoleic acid levels alone were the second and third most important determinants of PB, respectively. Meanwhile, in the 1-MCP treatment group, MDA content and CAT activity were found to be the second and third most important determinants of PB, respectively. FKP treatment increased the levels of aromatic compounds and delayed PB in flat peaches when compared with 1-MCP treatment. These results provide a theoretical basis for developing effective strategies to control PB and designing portable preservation methods for fruit and vegetable storage. In the future, by studying the influence of different fresh-keeping technologies on the flavor and quality of peaches during post-harvest refrigeration and storage, we hope to develop a safe, harmless, and convenient product for maintaining the freshness of this fruit and promoting the development of the peach industry.

## CRediT authorship contribution statement

**Xiaobin Ji:** Writing – review & editing, Writing – original draft, Supervision, Software, Resources, Project administration, Conceptualization. **Huijing Guo:** Validation, Resources, Project administration. **Jiluan Chen:** Validation, Resources, Methodology, Funding acquisition, Conceptualization. **Fangyuan Song:** Writing – original draft, Validation, Methodology, Investigation, Data curation, Conceptualization.

## Declaration of competing interest

The authors declare that they have no known competing financial interests or personal relationships that could have appeared to influence the work reported in this paper.

## Data Availability

Data will be made available on request.

## Appendix A Supplementary data

Supplementary material Image 1
